# Supplementary material for: Left ventricular trabeculation in Hominidae: divergence of the human cardiac phenotype
Source: Commun Biol. 2024 Jun 14;7:682. doi: 10.1038/s42003-024-06280-9 (PMC11178792; doi:10.1038/s42003-024-06280-9)
Supplement: Supplementary file 2 — Supplementary Information [file 42003_2024_6280_MOESM2_ESM.pdf]

Supplementary Materials

Left ventricular trabeculation in *Hominidae*: divergence of the human cardiac phenotype.

Bryony A. Curry, Aimee L. Drane#, Rebeca Atencia, Yedra Feltrer, Thalita Calvi, Ellie L. Milnes, Sophie Moittié, Annika Weigold, Tobias Knauf-Witzens, Arga Sawung Kusuma, Glyn Howatson, Christopher Palmer, Mike R. Stembridge, John E. Gorzynski, Neil D. Eves, Tony G. Dawkins, & Rob E. Shave#

#Corresponding author:

Rob Shave: rob.shave@ubc.ca

Aimee Drane: a.l.drane@swansea.ac.uk

This PDF file includes:

Supplementary Figures 1 to 3

Supplementary Tables 1 to 3

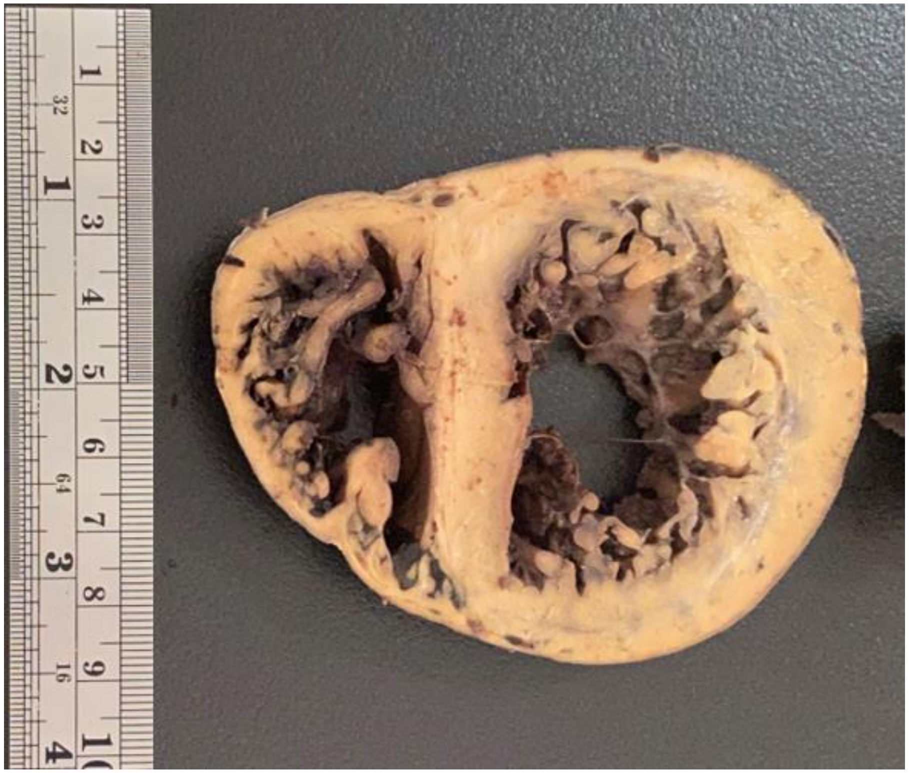

Supplementary Figure 1. Example image showing a transverse slice at the mid-level of the left ventricle in an adult chimpanzee (*Pan troglodytes*) post-mortem heart, demonstrating a high degree of trabeculation. A ruler is shown for scale, with measurements in both inches (left) and centimetres (right).

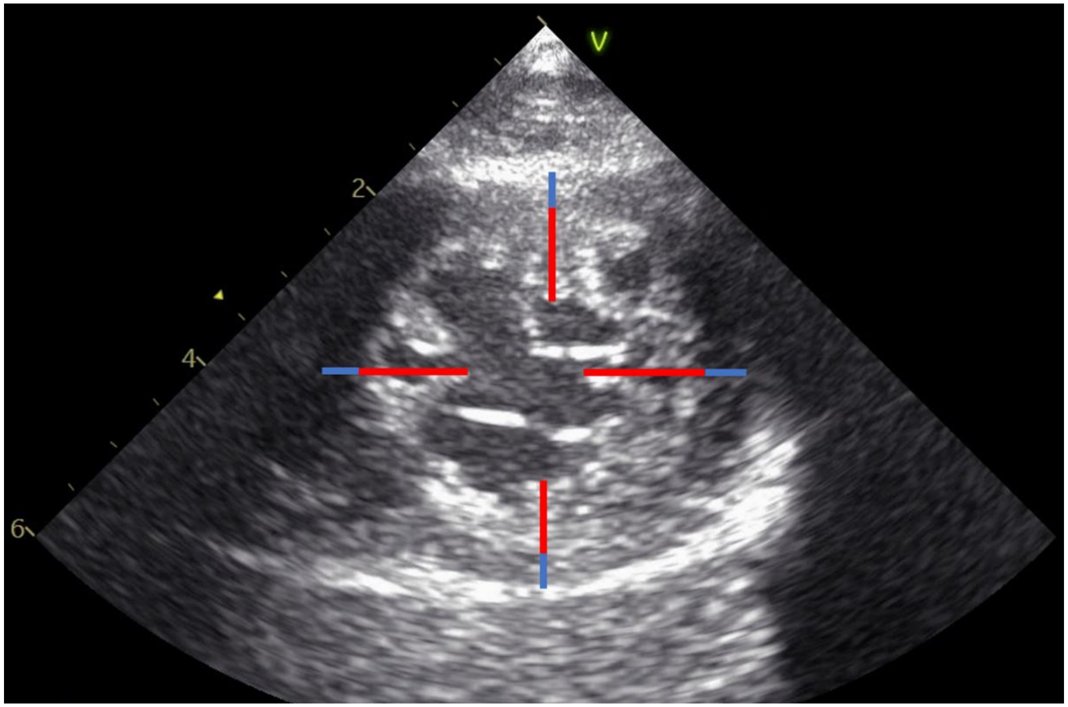

Supplementary Figure 2. A 2D echocardiographic example of a parasternal short-axis image acquired at the left ventricular apex, illustrating the measurement of the trabecular (red) and compact (blue) myocardium in a chimpanzee (*Pan troglodytes*).

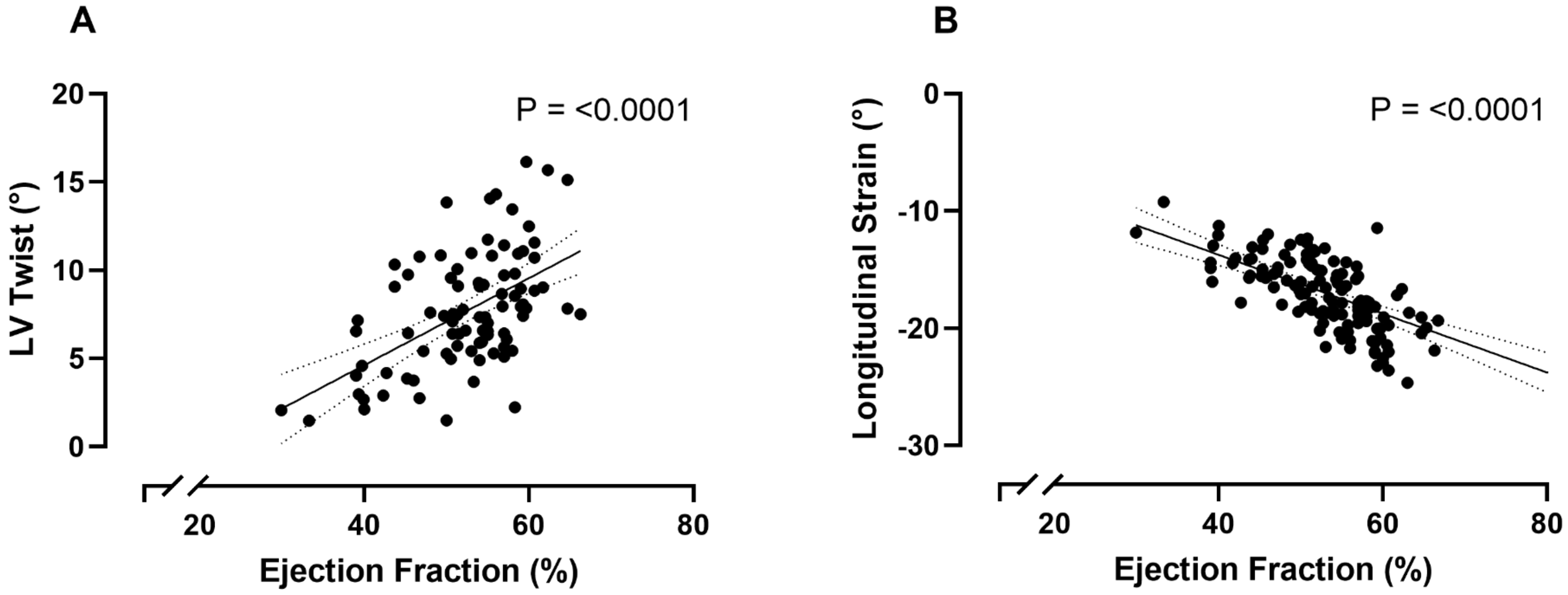

Supplementary Figure 3. The relationship between ejection fraction and peak left ventricular (LV) twist (A) (male *n* = 47, female *n* = 43) and peak longitudinal strain (B) (male *n* = 66, female *n* = 66) in adult male chimpanzees (*Pan troglodytes*). Linear regression lines are shown in solid black, with 95% confidence bands represented by the dotted line.

Supplementary Table 1. Left ventricular ratio of trabecular to compact myocardium in great apes. Trabeculation was assessed in accordance with the Jenni Criteria at end-systole, using short-axis echocardiographic images of the LV, in accordance with the 16-segment model recommended by the American Society of Echocardiography.

Variable	Humans (*n* = 34)	Chimpanzees (*n* = 242)	Bonobos (*n* = 7)	Gorillas (*n* = 14)	Orangutans (*n* = 28)
**Basal Segments**					
Anterior	0.38 ± 0.11*	0.62 ± 0.35‡	0.76 ± 0.36	0.92 ± 0.27	
Anterolateral	0.33 ± 0.09*	0.63 ± 0.42‡	1.10 ± 0.38	0.97 ± 0.42	
Inferolateral	0.33 ± 0.13*	1.05 ± 0.64	1.34 ± 0.29	1.48 ± 1.07	
Inferior	0.50 ± 0.58*	0.77 ± 0.36	1.01 ± 0.29	1.00 ± 0.38	
Inferoseptal	0.36 ± 0.13*	0.61 ± 0.29	0.74 ± 0.25	0.69 ± 0.29	
Anteroseptal	0.32 ± 0.14*	0.63 ± 0.38	1.59 ± 1.20	1.39 ± 1.08	
Average	0.34 ± 0.10* (0.21 – 0.61)	0.67 ± 0.23‡ (0.27 – 1.51)	0.95 ± 0.32 (0.56 – 1.45)	0.95 ± 0.24 (0.53 – 1.28)	
**Midpapillary Segments**					
Anterior	0.46 ± 0.18*	0.88 ± 0.39‡	1.14 ± 0.48	1.19 ± 0.32	
Anterolateral	0.46 ± 0.19*	0.75 ± 0.34‡	1.05 ± 0.63	1.13 ± 0.20	
Inferolateral	0.47 ± 0.21*	1.18 ± 0.75	1.90 ± 1.51	1.28 ± 0.57	
Inferior	0.44 ± 0.14*	0.85 ± 0.39	1.29 ± 0.78	1.20 ± 0.57	
Inferoseptal	0.43 ± 0.15†	0.64 ± 0.32	0.97 ± 0.42	0.69 ± 0.34	
Anteroseptal	0.34 ± 0.13*	0.93 ± 0.60	1.59 ± 0.80	1.25 ± 0.47	
Average	0.43 ± 0.13* (0.25 – 0.78)	0.83 ± 0.25 (0.38 – 1.76)	1.16 ± 0.30 (0.65 – 1.48)	0.90 ± 0.15 (0.59 – 1.24)	
**Apical Segments**					
Anterior	0.38 ± 0.12*	1.59 ± 0.68§	1.70 ± 0.48	1.71 ± 0.67	2.05 ± 0.78
Lateral	0.40 ± 0.17*	1.56 ± 0.63‡	1.60 ± 0.49	2.00 ± 0.41	1.99 ± 0.89
Inferior	0.43 ± 0.17*	1.78 ± 0.75	1.74 ± 0.69	1.99 ± 0.37	2.31 ± 0.94
Septal	0.45 ± 0.20*	1.20 ± 0.59‡	1.64 ± 0.47	1.74 ± 0.60	1.55 ± 0.78
Average	0.42 ± 0.15* (0.23 – 0.82)	1.46 ± 0.42‡§ (0.63 – 2.61)	1.58 ± 0.35 (1.17 – 2.28)	1.85 ± 0.34 (1.22 – 2.45)	1.94 ± 0.62 (1.04 – 4.10)

Trabecular:compact ratio for each left ventricular segment.

Data are presented as mean ± S.D. The range of the average ratio for the trabecular to compact myocardium for the levels of the mitral valve, midpapillary and apex is shown in brackets.

*$P < 0.05$ vs. all non-human great apes; $P < 0.05$ vs. chimpanzees and bonobos; ‡$P < 0.05$ vs. gorillas; §$P < 0.05$ vs. orangutans.

Supplementary Table 2. Left ventricular ratio of trabecular to compact myocardium in chimpanzees (*Pan troglodytes*) across age groups. Trabeculation was assessed in accordance with the Jenni Criteria at end-systole, using short-axis echocardiographic images of the LV, in accordance with the 16-segment model recommended by the American Society of Echocardiography. Infant ≤ 4 years; juvenile 5 - 7 years; sub-adult 8 - 12 years; and adult > 12 years of age.

Variable	Infant		Juvenile		Sub-Adult		Adult	
	Male (*n* = 11)	Female (*n* = 7)	Male (*n* = 14)	Female (*n* = 12)	Male (*n* = 22)	Female (*n* = 21)	Male (*n* = 79)	Female (*n* = 76)
**Basal Segments**								
Anterior	0.60 ± 0.25	0.72 ± 0.19	0.54 ± 0.31	0.56 ± 0.32	0.57 ± 0.17	0.72 ± 0.65	0.58 ± 0.24	0.66 ± 0.40
Anterolateral	0.62 ± 0.28	0.49 ± 0.22	0.67 ± 0.42	0.54 ± 0.23	0.58 ± 0.36	0.56 ± 0.26	0.63 ± 0.34	0.70 ± 0.59
Inferolateral	0.88 ± 0.42	0.91 ± 0.30	0.94 ± 0.33	0.84 ± 0.37	0.99 ± 0.56	1.00 ± 0.48	0.97 ± 0.54	1.10 ± 0.62
Inferior	0.76 ± 0.33	0.71 ± 0.21	0.59 ± 0.23	0.72 ± 0.28	0.83 ± 0.32	0.74 ± 0.32	0.74 ± 0.35	0.82 ± 0.41
Inferoseptal	0.65 ± 0.22	0.52 ± 0.14	0.53 ± 0.13	0.51 ± 0.22	0.55 ± 0.23	0.55 ± 0.30	0.65 ± 0.32	0.62 ± 0.31
Anteroseptal	0.61 ± 0.29	0.91 ± 0.36	0.46 ± 0.17	0.53 ± 0.28	0.66 ± 0.41	0.47 ± 0.18	0.61 ± 0.38	0.70 ± 0.43
Average	0.65 ± 0.19	0.69 ± 0.11	0.55 ± 0.13	0.61 ± 0.20	0.65 ± 0.17	0.61 ± 0.18	0.67 ± 0.24	0.71 ± 0.24
**Midpapillary Segments**								
Anterior	0.86 ± 0.20	1.01 ± 0.48	0.86 ± 0.36	0.79 ± 0.43	0.82 ± 0.30	0.81 ± 0.45	0.89 ± 0.37	0.88 ± 0.41
Anterolateral	0.64 ± 0.20	0.96 ± 0.41	0.87 ± 0.38	0.75 ± 0.34	0.73 ± 0.32	0.70 ± 0.39	0.78 ± 0.35	0.74 ± 0.35
Inferolateral	0.92 ± 0.54	1.35 ± 0.61	1.10 ± 0.48	1.23 ± 0.85	1.31 ± 0.60	0.96 ± 0.47	1.16 ± 0.80	1.24 ± 0.88
Inferior	0.76 ± 0.32	0.79 ± 0.24	1.03 ± 0.54	0.77 ± 0.37	0.84 ± 0.33	0.85 ± 0.33	0.90 ± 0.42*	0.77 ± 0.31
Inferoseptal	0.70 ± 0.21	0.58 ± 0.16	0.69 ± 0.27	0.68 ± 0.33	0.59 ± 0.30	0.60 ± 0.35	0.59 ± 0.32	0.61 ± 0.25
Anteroseptal	1.11 ± 0.66	0.96 ± 0.54	0.85 ± 0.47	1.04 ± 0.56	0.75 ± 0.53	0.85 ± 0.56	0.97 ± 0.56	0.92 ± 0.70
Average	0.77 ± 0.19	0.90 ± 0.27	0.86 ± 0.24	0.90 ± 0.37	0.79 ± 0.21	0.76 ± 0.25	0.83 ± 0.24	0.80 ± 0.23
**Apical Segments**								
Anterior	1.63 ± 0.46	1.24 ± 0.27	1.57 ± 0.65	1.44 ± 0.79	1.80 ± 0.65	1.75 ± 0.75	1.67 ± 0.68*	1.38 ± 0.59
Lateral	1.43 ± 0.40	1.31 ± 0.32	1.58 ± 0.71	1.53 ± 0.69	1.68 ± 0.68	1.64 ± 0.68	1.65 ± 0.63*	1.49 ± 0.62
Inferior	1.82 ± 0.85	1.28 ± 0.30	1.72 ± 0.62	2.02 ± 0.83	1.92 ± 0.88	1.60 ± 0.78	1.95 ± 0.74*	1.63 ± 0.69
Septal	0.91 ± 0.42	1.11 ± 0.49	1.46 ± 0.56	1.65 ± 0.97†	1.19 ± 0.61	0.95 ± 0.50	1.27 ± 0.61	1.11 ± 0.52
Average	1.40 ± 0.50	1.21 ± 0.17	1.55 ± 0.45	1.59 ± 0.61	1.57 ± 0.47	1.40 ± 0.49	1.56 ± 0.39*	1.33 ± 0.35

Trabecular:compact ratio for each left ventricular segment. Data are presented as mean ± S.D.

*$P < 0.05$; within-age group sex difference; †$P < 0.05$ vs. sub-adults.

Supplementary Table 3. Left ventricular rotation and deformation ('strain') comparison between adult humans and chimpanzees (*Pan troglodytes*).

	Males				Females			
		Chimpanzees		Humans		Chimpanzees		Humans
**Variable**	***n***		***n***		***n***		***n***	
**Twist Mechanics**								
Peak twist (°)	47	6.9 ± 3.1*† (5.9 – 7.8)	18	14.4 ± 2.7 (12.9 – 15.9)	43	8.5 ± 3.3* (7.5 – 9.5)	16	15.9 ± 4.0 (14.3 – 17.5)
Peak untwisting velocity (°sec-1)	47	-57.1 ± 26.0* (-65.1 – -49.1)	18	-100.1 ± 17.3* (-113.0 – -87.2)	43	-61.4 ± 27.2* (-69.8 – -53.1)	16	-109.6 ± 25.9 (-122.6 – -96.6)
Peak basal rotation (°)	59	-6.0 ± 2.1* (-6.6 – -5.5)	18	-4.9 ± 1.8 (-5.9 – -3.9)	55	-5.6 ± 2.0 (-6.2 – -5.1)	16	-5.2 ± 3.0 (-6.2 – -4.1)
Peak apical rotation (°)	59	2.6 ± 1.6*† (2.0 – 3.4)	18	10.0 ± 3.6 (8.8 – 11.3)	51	4.9 ± 2.9* (4.1 – 5.6)	16	11.5 ± 3.3 (10.2 – 12.8)
**Strain Mechanics**								
Peak basal radial strain (%)	59	28.0 ± 10.4* (25.0 – 31.1)	18	43.6 ± 11.3 (38.2 – 49.1)	55	31.9 ± 12.4* (28.8 – 35.0)	16	45.5 ± 14.2 (39.7 – 51.3)
Peak apical radial strain (%)	59	8.7 ± 7.1* (6.2 – 11.3)	18	25.3 ± 14.5 (20.7 – 29.8)	51	7.6 ± 9.1* (5.0 – 10.3)	16	27.0 ± 14.1 (22.1 – 31.8)
Peak basal circumferential strain (%)	59	-13.7 ± 3.3*† (-14.5 – -12.9)	18	-18.7 ± 2.7 (-20.1 – -17.2)	55	-16.3 ± 3.2* (-17.2 – -15.5)	16	-20.0 ± 2.4 (-21.5 – -18.4)
Peak apical circumferential strain (%)	59	-19.0 ± 3.2*† (-20.0 – -18.1)	18	-24.2 ± 4.5† (-25.9 – -22.5)	51	-22.6 ± 3.6* (-23.6 – -21.6)	16	-29.2 ± 3.7 (-31.0 – -27.4)
Peak longitudinal strain (%)	66	-15.0 ± 2.2*† (-15.6 – -14.5)	18	-18.8 ± 1.9† (-19.9 – -17.8)	66	-19.0 ± 2.4* (-19.6 – -18.5)	16	-22.6 ± 1.7 (-23.7 – -21.5)

Data are presented as mean ± S.D. 95% CI shown in brackets.

*$P < 0.05$; within-sex species difference; †$P < 0.05$; within-species sex difference.
