# Supplementary material for: Left ventricular trabeculation in Hominidae: divergence of the human cardiac phenotype
Source: Commun Biol. 2024 Jun 14;7:682. doi: 10.1038/s42003-024-06280-9 (PMC11178792; doi:10.1038/s42003-024-06280-9)
Supplement: Supplementary file 3 — Reporting Summary [file 42003_2024_6280_MOESM3_ESM.pdf]

nature portfolio

Corresponding author(s): Rob Shave

Last updated by author(s): Apr 10, 2024

Reporting Summary

Nature Portfolio wishes to improve the reproducibility of the work that we publish. This form provides structure for consistency and transparency in reporting. For further information on Nature Portfolio policies, see our Editorial Policies and the Editorial Policy Checklist.

Statistics

For all statistical analyses, confirm that the following items are present in the figure legend, table legend, main text, or Methods section.

n/a	Confirmed	
☐	☒	The exact sample size (*n*) for each experimental group/condition, given as a discrete number and unit of measurement
☐	☒	A statement on whether measurements were taken from distinct samples or whether the same sample was measured repeatedly
☐	☒	The statistical test(s) used AND whether they are one- or two-sided<br>*Only common tests should be described solely by name; describe more complex techniques in the Methods section.*
☒	☐	A description of all covariates tested
☐	☒	A description of any assumptions or corrections, such as tests of normality and adjustment for multiple comparisons
☐	☒	A full description of the statistical parameters including central tendency (e.g. means) or other basic estimates (e.g. regression coefficient) AND variation (e.g. standard deviation) or associated estimates of uncertainty (e.g. confidence intervals)
☐	☒	For null hypothesis testing, the test statistic (e.g. F, t, r) with confidence intervals, effect sizes, degrees of freedom and P value noted<br>*Give P values as exact values whenever suitable.*
☒	☐	For Bayesian analysis, information on the choice of priors and Markov chain Monte Carlo settings
☒	☐	For hierarchical and complex designs, identification of the appropriate level for tests and full reporting of outcomes
☐	☒	Estimates of effect sizes (e.g. Cohen's *d*, Pearson's *r*), indicating how they were calculated

Our web collection on statistics for biologists contains articles on many of the points above.

Software and code

Policy information about availability of computer code

Data collection	No software was used.
Data analysis	All statistical analyses were performed using the Statistical Package for the Social Sciences version 28 (SPSS Inc.) and GraphPad Prism (version 9.4.1 for Windows; San Diego, CA).

For manuscripts utilizing custom algorithms or software that are central to the research but not yet described in published literature, software must be made available to editors and reviewers. We strongly encourage code deposition in a community repository (e.g. GitHub). See the Nature Portfolio guidelines for submitting code & software for further information.

Data

Policy information about availability of data

All manuscripts must include a data availability statement. This statement should provide the following information, where applicable:

- Accession codes, unique identifiers, or web links for publicly available datasets
- A description of any restrictions on data availability
- For clinical datasets or third party data, please ensure that the statement adheres to our policy

The data that support the findings of this study are available in Figshare repository with the identifiers: https://doi.org/10.6084/m9.figshare.24274852 and https://doi.org/10.6084/m9.figshare.24274855.

Research involving human participants, their data, or biological material

Policy information about studies with human participants or human data. See also policy information about sex, gender (identity/presentation), and sexual orientation and race, ethnicity and racism.

Reporting on sex and gender	Sex was considered within the study design. Sex was determined based on self-reporting by human participants. Sex-based analyses were performed to determine whether the extent of trabeculation and left ventricular mechanics differed between sexes. Consent has been obtained for sharing of de-identified individual level data.
Reporting on race, ethnicity, or other socially relevant groupings	This study did not categorize human participants based upon race, ethnicity or other socially relevant groupings.
Population characteristics	Age, height and body mass have been reported in our human cohort of 18 males (mean age, 23.8 ± 2.8 years; body mass, 75.1 ± 8.5 kg; height, 179.8 ± 6.3 cm) and 16 females (mean age 22 ± 3.3 years; body mass 62.7 ± 8.3 kg; height 168.4 ± 7.3 cm).
Recruitment	Echocardiographic images of healthy human adult males and females were combined from previous studies for the human cohort (referenced in the manuscript). Briefly, human participants were recruited via poster advertisement around the local area, and through emails and social media. Interested individuals were sent a participant information sheet. Written, informed consent was obtained from all participants at the time of study enrollment, following a detailed explanation of experimental procedures.
Ethics oversight	The procedures and protocols involved in the collection of data from human participants were approved by the Institutional Clinical Research Ethics Board of the University of British Columbia (ethics approval numbers H12-03531 and H15-01513) and the Cardiff School of Sport and Health Sciences Research Ethics Committee (ethics approval number 17/3/01S). All procedures conformed to the ethical guidelines of the 1975 Declaration of Helsinki.

Note that full information on the approval of the study protocol must also be provided in the manuscript.

Field-specific reporting

Please select the one below that is the best fit for your research. If you are not sure, read the appropriate sections before making your selection.

☐ Life sciences ☐ Behavioural & social sciences ☒ Ecological, evolutionary & environmental sciences

For a reference copy of the document with all sections, see nature.com/documents/nr-reporting-summary-flat.pdf

Ecological, evolutionary & environmental sciences study design

All studies must disclose on these points even when the disclosure is negative.

Study description	Echocardiographic examinations were performed in all extant members of the Hominidae taxon to explore divergent evolution of the human left ventricle. Post-mortem analyses of chimpanzee hearts (n = 15) were used to corroborate our echocardiographic findings of a highly trabeculated ventricular wall in non-human great apes. Given the known relationship between cardiac form and function, we explored whether the highly trabeculated ventricular wall was related to mechanical indices of systolic and diastolic ventricular function. Across all great apes, a curvilinear relationship was present between the degree of trabeculation and LV twist during systole, and the velocity of ventricular untwisting during diastole, highlighting lower ventricular rotation in the trabeculated non-human great ape LV.
Research sample	The research sample consisted of all extant species of the Hominidae taxon, including:<br>1) 242 sanctuary-living chimpanzees (Pan troglodytes) (128 males, 115 females), aged 14.8 ± 7.5 years, range 1 – 36 years;<br>2) 28 sanctuary-living orangutans (Pongo) (16 males, 12 females) aged 10.4 ± 6.8 years, range: 1 – 25 years;<br>3) 14 zoological-living gorillas (6 males, 8 females) aged 24.2 ± 15.3 years, range 4 – 50 years;<br>4) 7 zoological-living bonobos (2 males, 5 females) aged 20.0 ± 14.8 years, range 7 – 46 years;<br>5) 34 humans; 18 males (mean age, 23.8 ± 2.8 years; body mass, 75.1 ± 8.5 kg; height, 179.8 ± 6.3 cm) and 16 females (mean age 22 ± 3.3 years; body mass 62.7 ± 8.3 kg; height 168.4 ± 7.3 cm).
Sampling strategy	No sample-size calculation was performed. Data were collected during pre-planned health assessments or routine veterinary procedures in great apes, which was at the discretion of the lead veterinarian of each sanctuary/zoological institution. Therefore, the sample size was opportunistic. This is the largest cohort of echocardiographic data on all extant great ape species to date.
Data collection	Transthoracic echocardiographic examinations in non-human great apes were performed by a single highly-trained sonographer (A.L.D). Three highly-trained sonographers (A.L.D, T.G.D, M.S) performed the echocardiographic examinations in the human cohort. All data were recorded offline and analyzed by a single researcher (B.A.C).
Timing and spatial scale	Complete cardiac examinations were performed during preplanned health assessments or routine veterinary procedures conducted between 2013 - 2019. The timing of the assessments were at the discretion of the lead veterinarian at each institution.

Data exclusions	The exclusion criteria was pre-established. Only those that were non-pregnant and presumed healthy based on physical and echocardiographic examinations were included in the study.
Reproducibility	The average intra-observer coefficient of variation for the measurement of compacted and trabeculated myocardium for all LV segments was 9%. The Intra-observer coefficient of variation for LV rotation and deformation within our group has been previously reported to be between 8 and 11% (see reference in manuscript).
Randomization	The allocation was not random. Chimpanzee and human data were split into groups based on sex, and chimpanzee data was split into groups based on age.
Blinding	Given the nature of this study, blinding of data acquisition is not possible. This study has been predicated upon data collected during health assessments at sanctuary/zoological institutions. As such, the echocardiographic images contain the associated meta-data for the purposes of generating institutional reports, meaning the animal ID cannot be blinded. However, the researcher who performed the analysis was blinded to the sex and age of the animal.

Did the study involve field work? ☒ Yes ☐ No

Field work, collection and transport

Field conditions	Echocardiographic examinations of chimpanzees were performed at one of three Pan African Sanctuary Alliance (PASA) member sanctuaries (Tchimpounga Chimpanzee Rehabilitation Centre, Congo; Chimfunshi Wildlife Orphanage, Zambia; Tacugama Chimpanzee Sanctuary, Sierra Leone. Echocardiographic examinations of orangutans were performed at the Nyaru Menteng Orangutan Rescue and Rehabilitation Center, Borneo, and assessments of gorillas and bonobos were performed in zoological institutions. Other field conditions are not relevant to the findings of this study.
Location	See above.
Access & import/export	Not applicable.
Disturbance	Not applicable.

Reporting for specific materials, systems and methods

We require information from authors about some types of materials, experimental systems and methods used in many studies. Here, indicate whether each material, system or method listed is relevant to your study. If you are not sure if a list item applies to your research, read the appropriate section before selecting a response.

Materials & experimental systems

n/a	Involved in the study
☒	☐ Antibodies
☒	☐ Eukaryotic cell lines
☒	☐ Palaeontology and archaeology
☐	☒ Animals and other organisms
☒	☐ Clinical data
☒	☐ Dual use research of concern
☒	☐ Plants

Methods

n/a	Involved in the study
☒	☐ ChIP-seq
☒	☐ Flow cytometry
☒	☐ MRI-based neuroimaging

Animals and other research organisms

Policy information about studies involving animals; ARRIVE guidelines recommended for reporting animal research, and Sex and Gender in Research

Laboratory animals	The study did not involve laboratory animals.
Wild animals	This study involved animals living in sanctuaries and zoological institutions. Care guidelines were followed in accordance with the Pan African Sanctuary Alliance and the European Association of Zoos and Aquaria best practice guidelines (as referenced in manuscript).
Reporting on sex	Sex was considered within the study design. Sex was determined by the lead veterinarian of each institution based on the external morphological phenotype. Sex-based analyses were performed to determine whether the extent of trabeculation and left ventricular mechanics differed between sexes, in the chimpanzee cohort. Sex-based analysis was not possible in the other non-human great ape species, owing to the small sample size. Therefore, left ventricular trabeculation was reported as an average of data from both sexes in gorillas, bonobos and orangutans.

Field-collected samples	The study did not involve samples collected from the field.
Ethics oversight	The procedures and protocols involved in the collection of non-human great ape data have been approved by, and adhered to, the PASA professional, ethical and welfare standards, endorsed by the British and Irish Association of Zoos and Aquariums, and ethically approved by the University of British Columbia, Canada (ethics approval number A23-0074).

Note that full information on the approval of the study protocol must also be provided in the manuscript.

Plants

Seed stocks	Not applicable.
Novel plant genotypes	Not applicable.
Authentication	Not applicable.
